# Consumption of cow's milk formula in the nursery and the development of milk allergy

**DOI:** 10.1002/clt2.12352

**Published:** 2024-04-12

**Authors:** Arnon Elizur, Shirel Rachel‐Jossefi, Marianna Rachmiel, Eli Eisenberg, Yitzhak Katz

**Affiliations:** ^1^ Shamir Medical Center Assaf Harofeh Pediatrics Division Institute of Allergy Immunology and Pediatric Pulmonology Tzrifin Israel; ^2^ Tel Aviv University School of Medicine Tel Aviv Israel; ^3^ Shamir Medical Center Assaf Harofeh Pediatrics Division Pediatric Endocrinology and Diabetes Institute Tzrifin Israel; ^4^ Tel Aviv University School of Physics and Astronomy Tal Aviv Israel

**Keywords:** breastfeeding, cow's milk, formula, milk allergy

## Abstract

**Background:**

The effect of the amount of transient cow's milk formula (CMF) consumed during the first days of life on IgE‐cow's milk allergy (IgE‐CMA) is unknown.

**Methods:**

A cohort of 58 patients with IgE‐CMA was identified from a large scale population‐based study of 13,019 infants followed from birth. A group of 116 infants matched for sex and breastfeeding only duration (beyond the nursery period), and another random group of 259 healthy infants were used as controls. Parents were interviewed and the infants' medical records were searched to assess CMF consumption in the nursery.

**Results:**

While 96% of the mothers of the 174 infants (58 with Cow's milk allergy and 116 controls) reported on exclusive breastfeeding during the stay in the nursery, CMF consumption was documented in 96 (55%) of the infants. Of those, most (57; 59%) received one to three feedings, 20 (21%) received four to nine feedings, and 19 (20%) received ≥10 feedings. Fewer formula feeds (1–3) were significantly more common in the allergic group than ≥4 feeds (*p* = 0.0003) and no feeds at all (*p* = 0.02) compared to controls (*n* = 116). Of those exclusively breastfed in the nursery, 13/23 allergic infants (57%) introduced CMF at age 105–194 days (the period with highest‐risk for IgE‐CMA) compared to 33/98 (34%) from the random control group (*n* = 259) (*p* = 0.04).

**Conclusions:**

Most infants end up receiving few CMF feeds in the nursery. Transient CMF in the nursery is associated with increased risk of IgE‐CMA.

## 1 INTRODUCTION

Cow's milk allergy (CMA) constitutes a global problem with increasing prevalence.^1^, ^2^ Although CMA spontaneously resolves in most patients, those with persistent CMA are at risk of severe and even fatal reactions and their quality of life is impaired.^3^, ^4^, ^5^, ^6^ While oral immunotherapy (OIT) has become a promising treatment for food‐allergic patients, milk allergy treatment is associated with more severe reactions and worse OIT outcomes.^7^, ^8^, ^9^ Therefore, primary prevention of milk allergy is of paramount importance.

The concept of early introduction of allergenic foods as a way to reduce the risk of IgE‐mediated food allergy is now widely accepted.^10^, ^11^, ^12^ Early introduction of several foods is currently recommended from age 4–6 months. In the case of milk, a large population‐based study of 13,234 infants with 98.4% catchment rate demonstrated that introduction of cow's milk protein (CMP) at 4–6 months was actually associated with the highest rate of IgE‐CMA. Importantly, milk allergy was rare among those with sustained milk introduction within the first 14 days of life.^13^ Observational case‐control studies and large population‐based studies on the timing of CMP introduction and the development of CMA were subsequently conducted with contradicting results.^14^, ^15^, ^16^, ^17^, ^18^, ^19^, ^20^ In addition, the known health benefits of breastfeeding and the recommendation of exclusive breastfeeding for the first 6 months of life further complicates this issue.^21^

Transient exposure to CMP in the first days of life might have an effect on the future development of CMA. Several studies have shown that early transient CMP consumption in the first days of life is associated with a higher rate of CMA^22^, ^23^ and the concept that early introduction has to be sustained to effectively prevent CMA is iterated in the 2021 EAACI guidelines^24^ and in an accompanying editorial.^25^ However, a recent meta‐analysis concluded that the evidence for timing of introduction of cow's milk and risk of CMA was very low certainty.^26^

The current study is based on data derived from a previous large cohort^13^ and analyzes actual documented CMP consumption in the nursery, its quantity, and its impact on the subsequent development of CMA.

## 2 METHODS

### 2.1 Study population

The feeding history of all 13,234 newborns born over a 2‐year period (between June 10, 2004 and June 30, 2006) at Shamir (Assaf Harofeh) Medical Center was collected, of which 13,019 infants completed the study (Figure S1).^13^ Any infant reported by his parents to have a possible adverse event related to CMP (*n* = 381) was invited to the allergy clinic for evaluation, as described in the supplementary methods. A total of 66 infants (0.5% of the study population) were diagnosed with IgE‐CMA based on a positive skin prick test (SPT) together with a history of a recent reaction to CMP or a positive oral food challenge.^13^ Forty‐eight (72.7%) patients fulfilled all criteria, including a suggestive history of an immediate response, a positive SPT response, and a positive challenge result to CMP. Seventeen patients did not perform an oral challenge. In 6 (9.1%) of these infants, an oral challenge was not offered because of life‐threatening responses to CMP exposure. In 11 infants, an oral challenge was not performed because of parental refusal. In a single case, the diagnosis was made by a private allergist, and by the time the infant was available for examination at the age of 9 months, the challenge result was negative. The most common symptoms of IgE‐CMA were cutaneous reactions (95.5%), including urticaria, angioedema, and pruritus, followed by gastrointestinal (54.6%) and respiratory (27.3%) symptoms. Of those 66 infants, eight patients were initially diagnosed as having food protein‐induced enterocolitis syndrome and only subsequently developed IgE‐CMA. These infants were classified as having secondary IgE‐CMA and excluded from the analysis, and the remaining 58 infants were analyzed. Additional 12,638 healthy infants, not having any complaints related to the ingestion of cow's milk formula (CMF) were identified. We used MATLAB's randperm function to randomly choose from this group 116 infants (for achieving 2:1 ratio) as the first control group (Figure S1). The purpose of this control group was to examine the effect of nursery consumption on IgE‐CMA by controlling for the effect of the long‐term milk exposure. We have shown in our previous work that IgE‐CMA risk was very low (0.05%) in infants who were introduced to CMP during the first 14 days, increased with CMP introduction at age 14–104 days, peaked at ages 105–194 days (1.75%), and then decreased again (0.5%). Similarly, the observed number of male‐allergic infants was somewhat larger than that of females. Therefore, this control group was selected while keeping the distribution of sex and period of CMF introduction matched with those of the allergic infants (Table S1). Other confounders, such as social class, pets, smoking habits, and atopic background, were not found to be significantly different between the control and IgE‐CMA groups in the original cohort.^13^ A second control group of 259 healthy infants was randomly selected to test the effect of nursery consumption of CMF on the association between breastfeeding duration and IgE‐CMA. Therefore, this group was not matched for the timing of CMF introduction or sex (Figure S1). The research protocol was approved by the Helsinki Review Board of the Assaf Harofeh Medical Center.

### 2.2 Feeding pattern during the nursery stay

Parents were asked about the frequency of CMF supplementation during their stay in the nursery in a telephone conversation 2–4 weeks after birth. In addition, for each infant in the IgE‐CMA group or the control groups, the nursery files were searched for actual documentation of breast‐milk and CMF feedings consumed by each infant in ml. Exclusive breastfeeding was defined as the consumption of breast milk only during the stay in the nursery, while breastfeeding only was defined as no consumption of CMF beyond the nursery period. In Israel, healthy full term newborns are discharged home 48–72 h after birth. Each feeding is recorded, providing data on both quantity and frequency of consumption. Nearly all (∼95%) 317 infants (58 with CMA and 259 controls) in the study were born full term and appropriate for gestational age, and were healthy. Therefore, according to the policy in Israel, these infants were likely discharged within 48–72 h, and the documentation of their feeding during this short time frame is likely accurate. No non‐dairy formula use in the nursery was recorded in the study.

### 2.3 Statistics

Two‐tailed Fisher's exact test with Yates continuity correction was used to analyze the relationship between categorical variables and odds‐ratio confidence interval, and a *t*‐test was used for comparing the number of feeds. Mann Whitney test was used to compare non‐normally distributed variables. *p*‐values *p* < 0.05 were considered significant; 95% odd‐ratios and confidence intervals are reported. The choice of which breastfeeding groups should be compared was dictated by our earlier results, and thus multiple‐testing corrections were ignored. Breastfeeding duration data were grouped into four categories following our previous findings.^13^ The number of formula feeds was grouped into three (roughly equally frequent) categories: no formula feeds, one to three formula feeds, four feeds or more. The power of our statistical tests was mostly dictated by the size of the (smaller) IgE‐CMA group. The randomly selected control groups were twice as large (or larger). Multivariate logistic regression: Data were checked for multicollinearity with the Belsley‐Kuh‐Welsch technique; Heteroskedasticity and normality of residuals were assessed by the White test and the Shapiro‐Wilk test, respectively.

## 3 RESULTS

**FIGURE 1:**
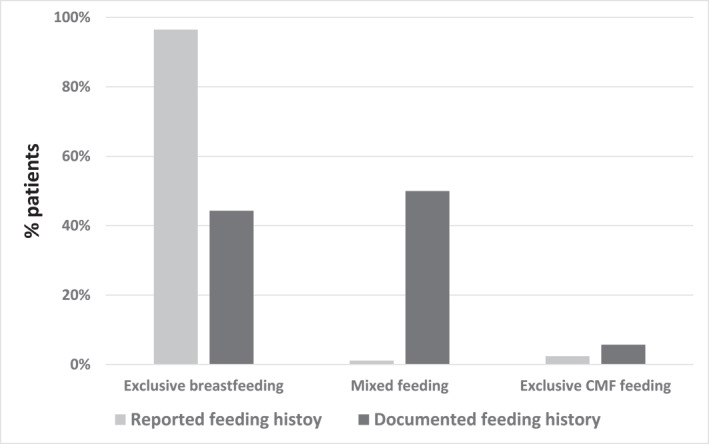
Reported and documented CMF feeding in the nursery. The reported and the documented feedings of CMF in the nursery in all 174 infants (58 infants with Cow's milk allergy and 116 controls). CMF, cow's milk formula.

**FIGURE 2:**
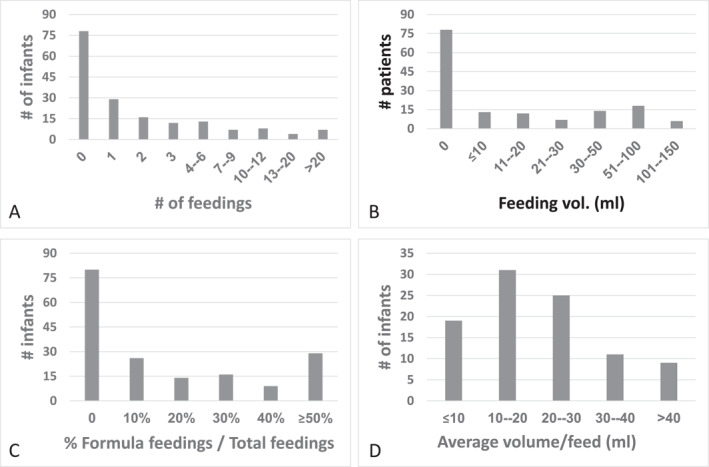
Number and volume of cow's milk formula feedings in the nursery. The number of feedings (A), their volume (B), the % of total feedings in the nursery given as formula (C), and the average volume of formula feedings (D) given to infants with cow's milk allergy (*n* = 58) and controls (*n* = 116) during the nursery stay.

Mothers of 167/174 (96%) infants (58 with IgE‐CMA and 116 of the first control group) reported that their newborn infant was exclusively breastfed during the stay in the nursery. In contrast, the documented feeding history showed that only 78 infants (44.8%) were truly not given CMF during that time (Figure 1). Only 5.5% of the mothers reported mixed breast and formula feeding in the nursery, while documented feeding history revealed that 56% of infants received mixed feeding. Non‐dairy formula was not used. Fifty‐seven infants (32.8%) received one to three feedings, 20 infants (11.5%) received four to nine feedings, and 20 infants (11.5%) received ≥10 feedings of CMF during their stay in the nursery (Figure 2A). The total amount of formula given to infants during their stay in the nursery was ≤20 mL in 26 infants, 20–50 mL in 38 infants, and >50 mL in 92 infants. The distribution of documented formula feeding in the nursery is shown in Figures 2B,C. The median formula volume per feeding was 20 mL (IQR = 14, 24), see Figure 2D.

**FIGURE 3:**
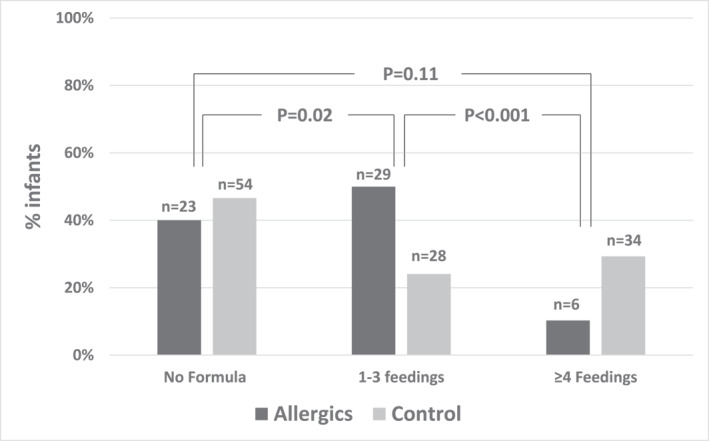
Formula feeding in the nursery based on allergic status. The number of formula feedings in the nursery (none, one to three feedings, ≥4 feedings) in infants with CMA (*n* = 58) versus controls (*n* = 116). For each of the three pairs of nursery feeding groups, the *p*‐value for the comparison of CMA and controls across these groups is presented. CMF, cow's milk formula.

The allergic and control groups were comparable in demographics and in perinatal history (Table 1). The fraction of infants that were not exposed at all to CMF was similar in the allergic (40%) and control groups (47%), respectively. Among those exposed, the median (IQR) number of formula feedings (2, one to three feedings) and the volume fed (30, 20–80 mL) given in the nursery to infants in the IgE‐CMA group was significantly lower compared to controls (4, 2–10 feedings, *p* = 0.005) and controls (78, 34–293 mL, *p* = 0.006), respectively. Given the differences in the number of formula feedings between allergic infants and controls, we next compared 1–3 versus ≥4 formula feedings between the two groups. There was a tendency toward a lower number of formula feedings (1–3 feedings) in the allergic infants (29/58, 50%) compared to only 28/116 (24%) in the control group (Table S2). Comparison to those who did not receive any CMF feedings (23/58 and 54/116, respectively) suggests a potential harmful effect for a small number of CMF feedings in the nursery. Among those infants who were exposed to CMF, only 6/35 (17%) of the allergic infants were given four formula feeds or more, compared to 34/62 (55%) of the control group (*p* = 0.0003 Fisher's exact test; OR = 5.8, 95% CI 2.0–19.4). Compared to infants who were not given formula at all, a similar or lower incidence of CMA was observed in those given four formula feeds or more (OR = 0.4, 95% CI 0.1–1.2; *p* = 0.11) (Figure 3). Applying multivariable regression (including gender as an explaining variable) reproduced the above results.

**TABLE 1:** Baseline characteristics of patients with CMA and controls.

Parameter	IgE‐CMA (*n* = 58)	Control 1 (*n* = 116)	Control 2 (*n* = 259)	*p* value (IgE vs. cont. 1)	*p* value (IgE vs. cont. 2)
Male sex	37 (63.8%)	73 (62.9%)	130 (50.4%)	1.0	0.08
CS delivery	9 (15.5%)	11 (9.5%)	32 (12.4%)	0.31	0.52
Urban residence	46 (79.3%)	98 (84.5%)	216 (83.7%)	0.4	0.44
Jewish ethnicity	55 (94.8%)	106 (93%)	230 (89.8%)	0.75	0.32
Gestat. Week^a^	39.5 (38–40)	40 (39–40)	40 (38–40)	0.24	0.82
Birth weight^a^	3.3 (3.0–3.5)	3.3 (3.0–3.6)	3.3 (3.0–3.5)	0.64	0.99
Maternal age^a^	29 (27–33)	29 (25–34)	29 (26–33)	0.86	0.83
Pat. Education^a^	14 (12–16)	14 (12–16)	13 (12–16)	0.79	0.84
Mat. Education^a^	14 (12–16)	14.5 (12–16)	13 (12–16)	0.67	0.48
Age at CMF introduction (d)^a^	120 (90–150)	120 (67–165)	30 (0–135)	0.69	<0.00001

^a^ Data presented in medians (IQR) and analyzed by Mann‐Whitney test.

Our next purpose was to examine differences in the duration of breastfeeding only, defined as no consumption of CMF following the nursery stay, as a function of formula feeding at the nursery (Table 2). Notably, 15 of the 29 allergic infants (52%) who were introduced to CMF at age 105–194 days, associated with the highest rate of CMA, received one to three feedings in the nursery. In comparison, only 12/58 (21%) of control infants introduced to CMF at these ages received one to three feedings (*p* = 0.006). The disparity is even more pronounced in infants who received at least one formula feed at the nursery and were later introduced to CMF at age 105–194 days. Of these, 15/16 allergic infants (94%) received one to three feedings, compared to 12/33 (36%) in the control group (*p* = 0.0002). The above results suggest that while consumption of a small number of formula feedings in the nursery is associated with an elevated risk for CMA, consumption of ≥4 formula feedings is not.

**TABLE 2:** Break‐up of allergic and 116 matched control infants according to breast feeding length and milk consumption at nursery.

Breastfeeding only duration (days)^a^	IgE‐CMA (58)			Matched controls (116)			*p*‐value
	No formula (*n* = 23)	1–3 formula (*n* = 29)	≥4 formula (*n* = 6)	No formula (*n* = 54)	1–3 formula (*n* = 28)	≥4 formula (*n* = 34)	
0–14	2 (66.7%)^b^	0	1 (33.3%)	2 (33.3%)	3 (50%)	1 (16.7%)	0.5
15–104	7 (33.3%)	11 (52.4%)	3 (14.3%)	20 (47.6%)	10 (23.8%)	12 (28.6%)	**0.04**
105–194	13 (44.8%)	15 (51.7%)	1 (3.4%)	25 (43.1%)	12 (20.7%)	21 (36.2%)	**0.0007**
>195	1 (20%)	3 (60%)	1 (20%)	7 (70%)	3 (30%)	0	**0.03**

*Note*: *p*‐values by Fisher's exact test for independence in the 3 × 2 contingency table, comparing (per row) the three values for IgE‐CMA versus the three values for the control group. Bolded values represent statistical significance (*p* < 0.05).

^a^ Refers to feeding beyond the nursery period.

^b^ Percentage represents rate with a specific row.

We then examined the risk of CMA development as a function of long‐term breastfeeding only for a given exposure of CMF at the nursery. We randomly selected 259 healthy infants from our cohort and retrieved their documented feeding history from the hospital nursery. The demographic and perinatal characteristics of this group were comparable to those of allergic patients (Table 1). For the 23 allergic infants not exposed to CMF in the nursery at all, only 2/23 (9%) began CMF consumption prior to 14 days of age, the period which is rarely associated with IgE‐CMA, and 13/23 (57%) were introduced to CMP at age 105–194 days (Table 3). In comparison, of the 98 healthy control infants not exposed at the nursery, 25/98 (26%) were exposed to CMF prior to 14 days of age, and 33/98 (34%) were introduced to CMP at age 105–194 days (OR = 4.9, CI = 1.0–23.8, *p* = 0.04) (Figure S2A). For infants exposed at the nursery for 1–3 times, none of the 29 allergic infants were later exposed to CMF prior to 14 days of age and 15/29 (52%) were introduced to CMP at age 105–194 days, compared to 24/56 (43%) and 11/56 (20%) of the healthy controls, respectively, suggesting that the elevated risk for late CMF introduction persists even if milk was consumed at the nursery (*p* < 0.0001) (Figure S2B). Since males are over‐represented in the IgE‐CMA group, we wanted to verify that this does not confound our results. Thus, we performed a logistic regression (*n* = 317; six explanatory variables: gender, three variables for breastfeeding groups 2,3,4 and two variables for 1–3 feedings and ≥4 feedings). The results are shown in Table S3, and support our previous results suggesting an increased risk for infants exposed to 1–3 feedings in the nursery as well as an increased risk for infants who are breastfed only for >14 days. Thus, the effect of cow milk consumption during the first months of life is observed even when one controls for the hospital nursery exposure (Figure S2C).

**TABLE 3:** Break‐up of allergic and randomly chosen control infants according to breast feeding length and milk consumption at nursery.

Breastfeeding only duration (days)^a^	IgE‐CMA (58)			Control (259)			*p*‐value
	No formula (*n* = 23)	1–3 formula (*n* = 29)	≥4 formula (*n* = 6)	No formula (*n* = 98)	1–3 formula (*n* = 56)	≥4 formula (*n* = 105)	
0–14	2 (8.8%)^b^	0	1 (16.7%)	25 (25.5%)	24 (42.8%)	72 (68.6%)	**0.002**
15–104	7 (30.4%)	11 (37.9%)	3 (50%)	28 (28.6%)	15 (26.8%)	20 (19%)	**0.006**
105–194	13 (56.5%)	15 (51.7%)	1 (16.7%)	33 (33.7%)	11 (19.6%)	8 (7.6%)	**0.000002**
>195	1 (4.3%)	3 (10.3%)	1 (16.7%)	12 (12.2%)	6 (10.7%)	5 (4.8%)	**0.03**

*Note*: *p*‐values by Fisher's exact test for independence in the 3 × 2 contingency table. Bolded values represent statistical significance (*p* < 0.05).

^a^ Refers to feeding beyond the nursery period.

^b^ Percentage represents rate within a specific column.

## 4 DISCUSSION

The current study demonstrates that most parents are unaware of their infants receiving small amounts of CMF in the nursery. Such exposure, followed by breastfeeding only, was associated with an increased risk of CMA. Moreover, the current study showed that while a small number of feedings was associated with an increased risk, a large number of feedings was not. Of note, even infants not exposed to CMP in the nursery seemed to be at increased risk of CMA if CMP introduction is delayed.

One major finding of this study was the increased occurrence of CMA in infants consuming small amounts of CMF (1–3 bottles) in the nursery compared with those receiving more feedings. Urashima et al found that sensitization to cow's milk was significantly higher among newborns randomized to consume ≥5 mL/d (suggesting that only a few doses of CMF were given) of CMF compared to those not receiving CMF for at least the first 3 days of life.^22^ Saarinen et al showed that supplementation with CMF (amount not specified) during the first 2 days after birth, followed by breastfeeding only or exposure to only small amounts of cow's milk at home during the first 8 weeks of life, was associated with increased risk of CMA, suggesting that both quantity and regularity of milk consumption are important.^27^, ^28^ Another study found that breastfed infants given formula supplements (amount not specified) at <24 h of age were seven times more likely than exclusively breastfed infants and 16 times more likely than exclusively bottle‐fed infants to exhibit CMA.^28^ It is likely that infants with mixed feedings received small amounts of formula, while those who were exclusively formula fed received large amounts.^29^ Taken together, these data suggest that the previously reported association between transient CMF feedings followed by prolonged exclusive breastfeeding and between IgE‐CMA depends on the actual amount of CMF feedings in the nursery. Several plausible explanations for this differential effect might be considered. First, the apparently protective effect of ≥4 feedings might reflect CMF consumption patterns later in life. Parents who are more tolerable toward larger amounts of nursery CMF exposure perhaps also tend to introduce CMF regularly earlier, before 6 months of age. However, this protective effect persisted even after controlling for breastfeeding duration only in the random control group. Second, it is possible that infants later to be diagnosed as allergic had some minor negative response to CMF feedings followed by early termination of CMF feedings in the nursery. In cases where care givers ignore these minor symptoms and continue CMF feedings, tolerance may be induced.

Most parents (>90%) in our study reported that their infants were exclusively breastfed in the nursery, while in fact <50% were, similar to a previous report.^30^ This suggests that most mothers are aware of the advantages of exclusive breastfeeding but have limited success in maintaining it. Nursery teams should be aware of the potential consequences of CMF feedings provided in the nursery, particularly in small amounts. In addition, the inaccuracy of parental reports should be acknowledged when assessing exclusive breastfeeding in the nursery.

Comparing only the group of children who were breastfed only up to 105–194 days (unmatched control group) showed an association with IgE‐CMA. A recent Israeli population‐based prospective study showed that the prevalence of CMA was 1.58% in exclusively breastfed infants, compared to zero in those receiving mixed or CMF feeding.^16^ However, protocol violations were frequent, and on per‐protocol analysis only 4/567 (0.7%) exclusively breastfed infants developed CMA. While the WHO recommends exclusive breastfeeding for at least 6 months, this duration is not reached in most cases, and CMP is introduced during the period of highest risk to develop CMA.^13^

This study has several limitations. First, the reported history of CMP consumption in the nursery was not collected during the nursery stay, and a recall bias might have been introduced. However, the history was taken only a few weeks after delivery. Also, this study represents the findings of a single Israeli center. While it is important to examine these findings in other countries, it should be acknowledged that such studies are difficult to perform. In addition, the study represents feeding practices from 2006. However, surveys conducted by the Israeli ministry of health in 2009 and 2019 demonstrate that transient CMF feeding in the nursery, followed by breastfeeding only, became more prevalent with time. Also, confounders, such as social class, pets, smoking habits, and atopic background, were not adjusted for in the current analysis as they were not found to be significantly different between the control and IgE‐CMA groups in the original cohort. Lastly, given that 54/58 patients developed CMA before 6 months of age, the recommended time for introducing complementary feeding, the effect of complementary feeding was unlikely to affect CMA in this cohort and was not examined.

In summary, we demonstrate that the consumption of small amounts of CMF in the nursery followed by CMF elimination is associated with increased risk of IgE‐CMA. Nursery staff should be aware of this effect as many parents are unaware of CMF consumption by their infants in the nursery.

## AUTHOR CONTRIBUTIONS

Yitzhak Katz and Eli Eisenberg contributed to the conception and design of the study, acquisition of data, analysis and interpretation of data, and drafting the manuscript. Arnon Elizur contributed to the analysis and interpretation of data, and drafting the manuscript. Shirel Rachel‐Jossefi and Marianna Rachmiel contributed to the acquisition of data, and drafting the manuscript.

## CONFLICT OF INTEREST STATEMENT

All authors have no conflicts of interest to disclose.

## Supporting information

Supporting Information S1

Figure S1

Figure S2

## Data Availability

Data will be available upon request.
