# Supplementary material for: Consumption of cow's milk formula in the nursery and the development of milk allergy
Source: Clin Transl Allergy. 2024 Apr 12;14(4):e12352. doi: 10.1002/clt2.12352 (PMC11015055; doi:10.1002/clt2.12352)
Supplement: Supplementary file 1 — Supporting Information S1 [file CLT2-14-e12352-s002.docx]

**SUPPLEMENTARY METHODS**

Study population

All newborns (13,234) born from June 10, 2004, to June 30, 2006, at the Assaf-Harofeh Medical Center were enrolled. Mothers were asked to contact the allergy clinic immediately after any adverse reaction suspected to be related to the initiation of CMP-based feeding or, in the lack of any unusual event, 14 to 30 days after the initiation of CMP-based feeding. The mothers were supplied with a kit containing an explanatory letter about the project, a prestamped envelope, and a card with contact details. An explanatory letter about the project was distributed to all health care providers in the region. If the parents did not contact the clinic by the age of 3 months, a telephone or mail contact was established, and the questionnaire was provided. The questionnaire requested demographic details; the length of exclusive breastfeeding, the age of introduction of CMP-based formula on a regular basis (at least once daily); and whether any adverse responses to CMP were noted. If the infant was still breast-fed at the time of the contact, the mother was encouraged to continue breast-feeding, and contacts were maintained at 2-month intervals until the infant started to consume CMP. Any parent noting a possible adverse event related to CMP

was interviewed and their infants were invited for an examination. In the clinic, the patient was examined and an SPT and an open challenge were offered, unless clinically contraindicated. Each final diagnosis of milk allergy was made independently by 2 of the study investigators. Cases of disagreement (2 cases) were resolved in a conjoint discussion.

Infant evaluation -

Skin prick tests (SPTs) to CMP, histamine (ALK-Abelló, Port Washington, NY) and a negative control were performed on the volar surface of the forearm and read after 20 minutes. A wheal of ≥3-mm or larger was considered positive (13). Oral food challenge (OFC) to cow's milk formula (CMF) was performed using Materna (Maabarot Products Ltd, Maabarot, Israel). Increasing doses were given, from a 1:10 diluted formula of 1.0 ml (2.7 mg of CMP) up to 120 ml (3.24 g of CMP) every 30 minutes. The challenge was terminated if a cutaneous, respiratory, gastrointestinal, or systemic response was observed. In case of a negative challenge result, the infant was observed for 3 hours, and a subsequent contact was made 2 weeks later to inquire about its feeding habits.

**Figure S1.** **A consort diagram of the study population**. A consort diagram demonstrating the initial cohort derived from the former study, and the groups (IgE-CMA and two control groups of the present study. 8 patients who were initially diagnosed as having food protein induced enterocolitis syndrome and only subsequently developed IgE-CMA, were classified as secondary IgE-CMA and were excluded from the analysis.

**Figure S2**. **Age of exposure to CMF beyond the nursery period, among allergic and control infants based on formula feeding in the nursery.** The percentage of infants introduced to CMF beyond the nursery period at <14 days or at 105-194 days among those who received no formula feedings in the nursey (A) and those who received 1-3 formula feedings in the nursey (B) and the percentage of IgE-CMA among the entire cohort of 13,019 infants and those who received no formula feedings in the nursey (extrapolated from control group 2 (n=259(, based on age at CMP introduction (C). The P-value shown are for 2x2 comparisons of the indicated groups between CMA and controls.

SUPPLEMENTARY TABLES

| **Control** | | | **IgE CMA** | | | **Breast-feeding only duration (days)^*^** |
| --- | --- | --- | --- | --- | --- | --- |
| **F**  **(n=43)** | **M**  **(n=73)** | **All**  **(n=116)** | **F**  **(n=21)** | **M**  **(n=37)** | **All**  **(n=58)** |  |
| 2 | 4 | 6 | 1 | 2 | 3 | 0-14 |
| 18 | 24 | 42 | 9 | 12 | 21 | 15-104 |
| 19 | 39 | 58 | 9 | 20 | 29 | 105-194 |
| 4 | 6 | 10 | 2 | 3 | 5 | >194 |

**Table S1.** **Distribution of sex and breast-feeding duration in the allergic infants and the control group**

**^*^** Refers to feeding beyond the nursery period

**Table S2**: Break-up of allergic and 116 matched control infants according to the number of formula feedings at nursery.

| **Number of formula feedings** | **IgE-CMA (n=58)** | **Matched Controls (n=116)** | **%IgE-CMA^*^** |
| --- | --- | --- | --- |
| 0 | 23 (39.7%) | 54 (46.6%) | 30% |
| 1 | 15 (25.8%) | 14 (12.1%) | 52% |
| 2 | 6 (10.3%) | 10 (8.6%) | 38% |
| 3 | 8 (13.8%) | 4 (3.4%) | 67% |
| 4 | 0 | 7 (6%) | 0% |
| 5 | 0 | 3 (2.6%) | 0% |
| 6 | 2 (3.4%) | 1 (0.9%) | 67% |
| 7 | 0 | 4 (3.4%) | 0% |
| 8 | 0 | 1 (0.9%) | 0% |
| 9 | 0 | 2 (1.7%) | 0% |
| 10 | 1 (1.7%) | 3 (2.6%) | 25% |
| >10 | 3 (5.2%) | 13 (11.2%) | 19% |

**^*^**Represents the % of IgE infants among all infants in a row

**Table S3. Logistic regression analysis for IgE-CMA and 2^nd^ Control group (n=317)**

| **Variable** | **Odds-ratio** | **p-value** |
| --- | --- | --- |
| BF only duration 15-104 days**^*^** | 10.97 [3.07;39.25] | **0.000229** |
| BF only duration 105-194 days**^*^** | 16.92 [4.71;60.82] | **1.48e-05** |
| BF only duration >194 days**^*^** | 7.04 [1.51;32.86] | **0.013** |
| 1-3 formula feedings in nursery | 2.75 [1.38;5.47] | **0.00408** |
| >3 formula feedings in nursery | 0.567 [0.207;1.55] | 0.268 |
| Gender (male) | 1.45 [0.754;2.78] | 0.267 |

**^*^** Refers to feeding beyond the nursery period
