# Supplementary figures and images for: Consumption of cow's milk formula in the nursery and the development of milk allergy
Source: Clin Transl Allergy. 2024 Apr 12;14(4):e12352. doi: 10.1002/clt2.12352 (PMC11015055; doi:10.1002/clt2.12352)

Figure S1

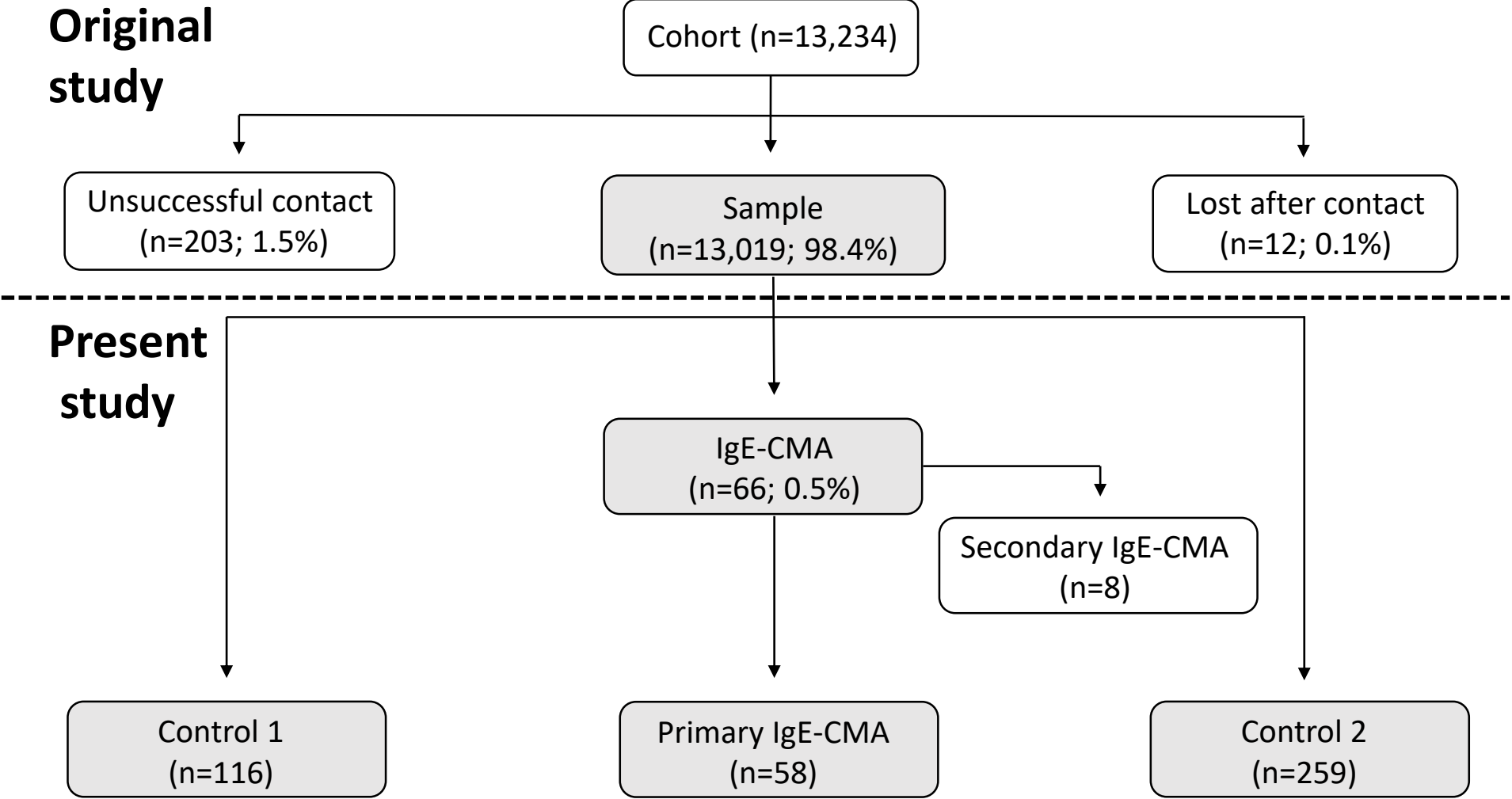

Supplement: Supplementary file 2 — Figure S1 [file CLT2-14-e12352-s001.pdf]

Figure S2

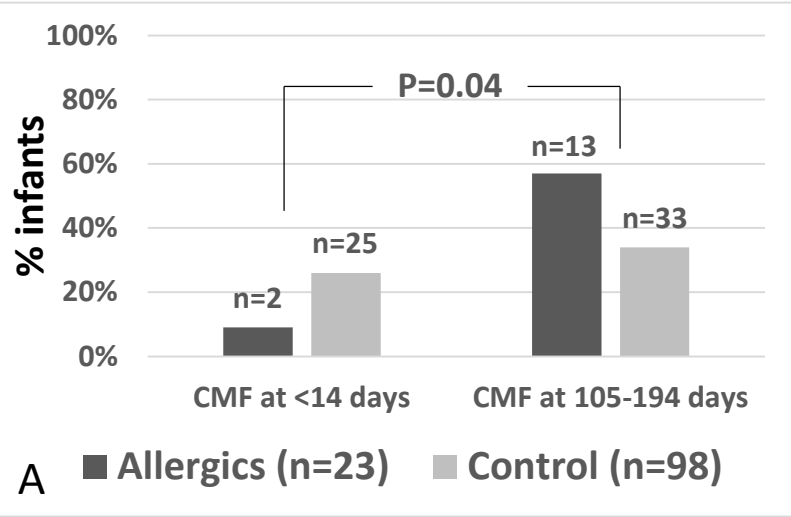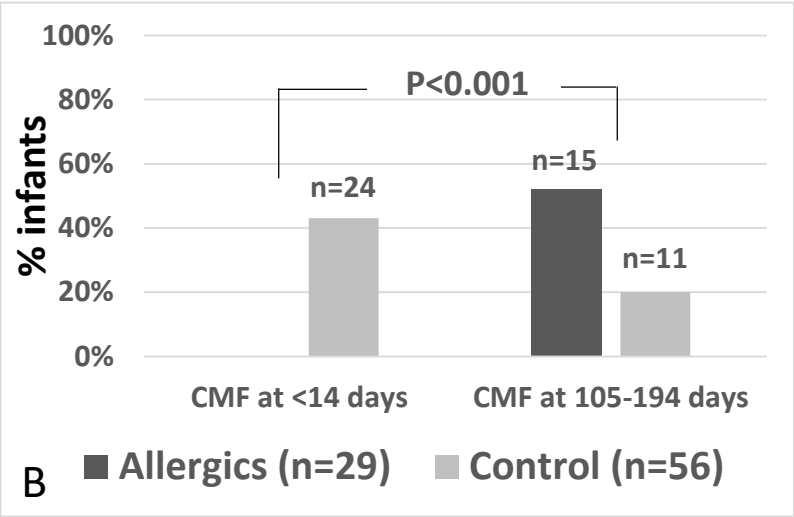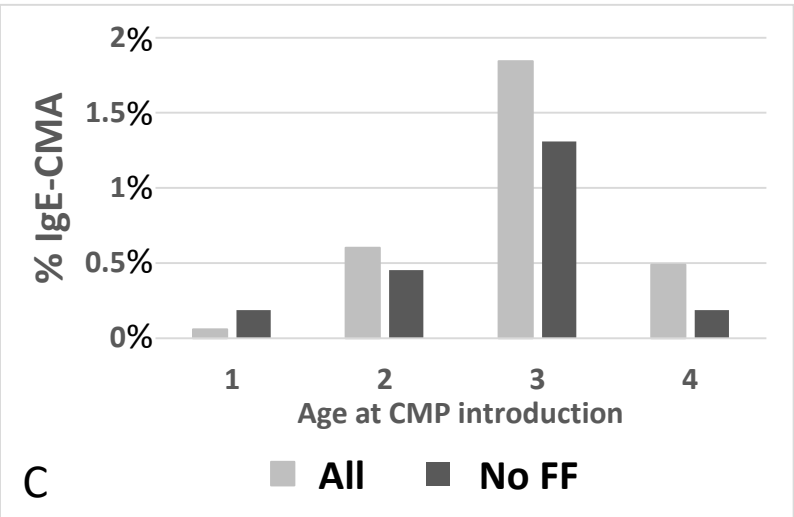

Supplement: Supplementary file 3 — Figure S2 [file CLT2-14-e12352-s003.pdf]
